# Comparison of Imputation Strategies for Incomplete Electronic Health Data

**DOI:** 10.34133/hds.0493

**Published:** 2026-11-02

**Authors:** Shuo Zhang, Zhilong Zhang, Yuxi Zhou, Huixin Liu

**Affiliations:** ^1^ Department of Computer Science, Tianjin University of Technology, Tianjin, China.; ^2^ National Institute of Health Data Science, Peking University, Beijing, China.; ^3^ DCST, BNRist, RIIT, Institute of Internet Industry, Tsinghua University, Beijing, China.; ^4^ Department of Clinical Epidemiology and Biostatistics, Peking University People’s Hospital, Beijing, China.

## Abstract

**Background:** Missing data are a common challenge in clinical tabular datasets and can substantially affect both statistical analyses and predictive modeling in healthcare. **Methods:** In this study, we conducted an empirical evaluation of 8 imputation strategies—median imputation, median imputation with missing indicator, multiple imputation by chained equations (MICE), MissForest, generative adversarial imputation network (GAIN), missing data imputation using autoencoders (MIWAE), self-attention-based imputation for time series, and gated recurrent unit with decay—across 3 heterogeneous real-world clinical datasets. The methods were assessed under controlled missingness simulations covering different mechanisms (missing completely at random, missing at random [MAR], and missing not at random [MNAR]) and missingness rates ranging from 10% to 90%. We evaluated imputation quality using multiple statistical metrics and examined its relationship with downstream classification performance across multiple classifiers. **Results:** Across the considered settings, MICE and MissForest showed consistently strong and stable performance, while deep generative approaches such as GAIN and MIWAE exhibited higher variability, particularly under the MAR and MNAR mechanisms and at higher missingness rates. Importantly, we observed that improvements in imputation accuracy did not necessarily translate into better downstream predictive performance. **Conclusion:** Rather than drawing general conclusions about method superiority, the results highlight the sensitivity of imputation outcomes to data characteristics, missingness mechanisms, and evaluation objectives. These findings underscore the importance of aligning imputation strategy selection with the intended downstream task and data regime when working with heterogeneous clinical tabular data.

## Introduction

Missing data are a pervasive issue in electronic health records and pose a major challenge for clinical research and digital health applications [1,2]. Such missingness can arise from various sources, including incomplete data collection, privacy constraints, heterogeneous data integration, and selective clinical testing. These factors contribute to complex and often nonrandom missing data patterns. According to the widely accepted taxonomy, missingness can occur under 3 mechanisms: missing completely at random (MCAR), missing at random (MAR), and missing not at random (MNAR) [3]. In clinical datasets, true MCAR is rare; missingness is more frequently driven by observed or unobserved covariates, resulting in MAR or MNAR mechanisms [4,5]. For instance, in intensive care settings, critically ill patients may be unable to undergo certain tests, leading to systematic missingness in key clinical variables, while less severe cases are more fully documented. These patterns are often correlated with disease severity, and inappropriate imputation strategies may introduce bias, ultimately compromising model performance and clinical decision-making.

To address missing data in tabular settings, a wide range of imputation strategies has been proposed [5]. Classical approaches, including mean or median imputation, multiple imputation by chained equations (MICE) [6], and MissForest [7], are widely used in practice due to their simplicity and interpretability. More recently, deep-learning-based approaches, such as generative adversarial imputation network (GAIN) [8] and variational-autoencoder-based methods, including missing data imputation using autoencoders (MIWAE) [9], have been introduced to model complex feature dependencies and missing data patterns. More recent developments include self-attention-based methods such as self-attention-based imputation for time series (SAITS) [10] and recurrent architectures such as gated recurrent unit with decay (GRU-D) [11], which were originally designed for time-series data but have also been applied to clinical tabular settings. These methods are motivated by their capacity to capture nonlinear relationships in high-dimensional data and have been evaluated across a variety of synthetic and real-world datasets. The empirical behavior of such methods in heterogeneous clinical tabular data, however, can depend on multiple factors, including data characteristics, missingness mechanisms, and evaluation objectives.

Previous work has shown that imputation methods are often evaluated primarily in terms of reconstruction accuracy. However, a growing body of evidence suggests that improvements in imputation accuracy do not necessarily translate into better downstream predictive performance, particularly when missingness is informative or related to outcomes and covariates [12]. In addition, although the MCAR assumption is frequently adopted for methodological convenience, missingness mechanisms in clinical data are more realistically characterized by MAR or MNAR processes, which can substantially influence both imputation behavior and downstream analyses [13]. As a result, there remains ongoing debate regarding which imputation strategies are most appropriate under different missingness mechanisms and levels, as well as how imputation quality should be assessed in the context of clinical prediction tasks [14]. Moreover, practical guidance is still limited with respect to identifying missingness regimes beyond which imputation may become unreliable or of limited utility for downstream modeling.

Recent work has begun to systematically benchmark missing-data-handling strategies in the context of predictive modeling using health databases. For instance, the study by Perez-Lebel et al. [15] evaluated missing-value imputation approaches in predictive modeling across multiple healthcare datasets and demonstrated that the relative effectiveness of different strategies can vary with datasets, prediction tasks, and missingness settings. A comprehensive comparison across multiple missingness mechanisms and downstream tasks was presented in work by Zhou et al. [16]; however, this evaluation was limited to general-purpose datasets with only numerical variables, leaving clinical tabular data with mixed variable types relatively underexplored. More broadly, existing benchmarking efforts have largely focused on general-purpose datasets or restricted missingness scenarios, without a systematic investigation of the full spectrum of missingness mechanisms and rates on real-world clinical tabular data. Additionally, although recent studies have emphasized the value of task-oriented evaluation [17], the consistency of downstream predictive performance across different classifier families remains understudied in clinical applications.

To address these limitations, this study provides a systematic benchmark of 8 representative imputation strategies on heterogeneous real-world clinical tabular datasets, evaluating both imputation quality and downstream predictive utility across multiple missingness mechanisms, missingness rates, variable types, and downstream classifiers. Our goal is to provide empirically grounded insights into the behavior of commonly used imputation strategies under clinically relevant missingness settings and to support more informed preprocessing choices in clinical machine learning applications.

## Methods

### Experimental design

This study followed a systematic empirical evaluation framework to assess the performance of imputation methods on clinical tabular data. The overall workflow comprised 4 main stages: (a) data acquisition and preprocessing of 3 heterogeneous clinical datasets (MIMIC-III [Medical Information Mart for Intensive Care III], Breast Cancer, and Kaggle COVID-19); (b) simulation of missing data under 3 mechanisms (MCAR, MAR, and MNAR) with varying missingness rates (10% to 90%); (c) application of 8 imputation strategies spanning simple statistical methods, machine-learning-based approaches, and deep generative models; and (d) dual-aspect evaluation focusing on both imputation accuracy and downstream predictive utility across multiple classifiers, with XGBoost native missing-value handling included as a no-imputation reference baseline.

### Dataset

In this study, we focus on 3 real-world clinical datasets: MIMIC-III, Breast Cancer, and Kaggle COVID-19.

MIMIC-III is a publicly available critical care database containing de-identified clinical data from patients admitted to the intensive care units (ICUs) of Beth Israel Deaconess Medical Center between 2001 and 2012 [18]. The subset used in this study was derived from a preprocessing pipeline adapted from Shadbahr et al. [17]. We restricted the cohort to patients’ first ICU admission, aged over 15, with at least 3 d of ICU stay and emergency or urgent admission type (21,812 patients). Seven clinical variables recorded in more than 50% of patients were extracted, and the mean and standard deviation of each variable over the first 10 ICU days were computed per patient, yielding 14 numerical features with no missing values by design. A random subset of one-third of patients (7,214 patients) was used in this study.

The Breast Cancer dataset originates from oncology data collected at Memorial Sloan Kettering Cancer Center (MSKCC) between April 2014 and March 2017 [19]. It includes genomic analysis data for 1,918 tumor samples, with detailed clinical variables, treatment information, and outcomes for 1,756 patients. For this study, we used a preprocessed version containing 16 key features and retained the natural missingness in the data to more accurately evaluate the performance of different imputation methods on real-world medical data.

The Kaggle COVID-19 dataset is sourced from clinical data collected at a tertiary care hospital in São Paulo, Brazil. This dataset includes clinical records for COVID-19 patients and simulates different missing data patterns to explore the applicability of missing-data-handling methods for pandemic-related medical data. It is characterized by substantial heterogeneity and dynamic changes, making it suitable for validating the generalization capabilities of imputation methods across various types of medical data. The version used in this study contains data from 1,925 patients with 19 key features.

Use of all datasets complied with their respective access and usage guidelines.

### Data preprocessing

The MIMIC-III dataset consists solely of numerical variables. In contrast, the Breast Cancer and COVID-19 datasets include numerical, categorical (both nominal and ordinal), and multilevel variables. Before analysis, categorical variables were transformed using one-hot encoding, while ordinal variables were encoded as integers. This ensured compatibility across imputation and modeling pipelines.

### Outcome variables

For the MIMIC-III, Breast Cancer, and Kaggle COVID-19 datasets, we used survival status as the outcome variable of interest. Survival status was chosen as the primary outcome to reflect clinically meaningful endpoints across the 3 datasets, formulated as a binary classification task.

### Missingness simulation

In this experiment, we simulated missingness in the 3 datasets mentioned above using 3 different missing data mechanisms: MCAR, MAR, and MNAR. For MCAR, we custom-built a function to generate missing data. Specifically, we randomly generated a mask matrix matching the dimensions of the data, where the values were determined by a preset missing rate to decide whether to assign a missing value (NaN) to each data point. The missing rate varied from 10% to 90% by 10%.

For MAR and MNAR, we used the pyampute package to automatically generate missing data [20]. For each experimental run, missing variables were randomly selected as half of all available features. A single amputation pattern was applied using MultivariateAmputation with the specified mechanism and missingness rate. Under MAR, missingness probability was determined by a logistic function of the remaining observed variables with equal weights (pyampute default settings), representing scenarios where the probability of missingness depends on other observed clinical measurements. Under MNAR, missingness probability was determined by a logistic function of each variable’s own observed values (pyampute default settings), representing situations where severely ill patients may be unable to undergo certain tests due to their condition. This procedure was applied independently to both training and test sets. The missing rate was set to range from 10% to 90% in increments of 10%.

### Imputation methods

In this study, we evaluated 8 imputation methods spanning a range of methodological complexity. Classical methods include median imputation, MICE, and MissForest. Deep-learning-based methods include GAIN and MIWAE. To incorporate more recent deep learning approaches, we additionally included SAITS [10] and GRU-D [11]. As SAITS and GRU-D are originally designed for time-series data, they were adapted for use on cross-sectional tabular data by reshaping each sample into pseudo-time steps (n_steps = 2 for SAITS) or treating each sample as a single time step with uniform time intervals (*seq_len* = 1 for GRU-D). While this adaptation limits their ability to exploit temporal dynamics, it allows for a broader comparison of deep learning imputation architectures in the clinical tabular setting. In addition, we included median imputation with missing indicator (Median+Indicator) as an augmented classical baseline. This method extends standard median imputation by appending a set of binary indicator variables to the feature matrix, where each indicator flags whether the corresponding value in a given sample was originally missing. This allows downstream classifiers to explicitly leverage missingness patterns as predictive signals, potentially improving classification performance when missingness is informative. Unlike standard median imputation, which discards information about the location of missing values, Median+Indicator retains this structural information at the cost of an expanded feature space.

### Hyperparameter configuration

All methods were evaluated using fixed, pre-specified hyperparameters without any dataset-specific tuning, ensuring a fair and reproducible comparison across methods.

Classical methods were implemented using scikit-learn: MICE (IterativeImputer, max_iter = 10) and MissForest (IterativeImputer with RandomForestRegressor, default settings).

GAIN was implemented following Yoon et al. [8] with a 3-layer multilayer perceptron (MLP) for both the generator and discriminator, with hidden size equal to the input feature dimension. The training parameters are batch size = 128, hint rate = 0.9, alpha = 10, iterations = 3,000, and learning rate = 1e−5 (Adam optimizer).

MIWAE was implemented following Mattei and Frellsen [9] with a 3-layer MLP encoder (output dimension = 2× latent dimension) and a 3-layer MLP decoder (output dimension = 3× input features, parameterizing a Student *t* distribution). The training parameters are hidden units = 128, batch size = 64, learning rate = 0.001 (Adam), epochs = 100, latent dimension = 20, and importance samples *K* = 20.

SAITS was implemented using the PyPOTS library [10]. As SAITS is originally designed for time-series data, tabular samples were adapted by reshaping each sample into *n_steps* = 2 pseudo-time steps to satisfy the required input format. The model parameters are *n_layers* = 2, *d_model* = 64, *d_ffn* = 128, *n_heads* = 4, *d_k* = 16, *d_v* = 16, batch size = 128, epochs = 100, patience = 10, and learning rate = 1e−4 (Adam optimizer).

GRU-D [11] was adapted for tabular data by treating each sample as a single time step (*seq_len* = 1), with time decay intervals (∆) set uniformly to 1 to accommodate the absence of a real temporal structure. Missing values were initialized to the feature-wise mean computed from training data. The model parameters are hidden size = 64, batch size = 64, epochs = 50, and learning rate = 1e−3 (Adam optimizer).

### Classification model

To assess the utility of the imputed data in predictive tasks, we trained 2 classifiers on each completed dataset after imputation: random forest (RF) and logistic regression (LR). RF is a robust ensemble learning method known for its effectiveness in handling high-dimensional, heterogeneous clinical data and its resistance to overfitting [21–23]. LR serves as a linear baseline classifier widely used in clinical prediction tasks. The use of multiple downstream classifiers allows us to assess whether the relationship between imputation quality and predictive performance is consistent across different model families, rather than being specific to a single classifier. Additionally, XGBoost with native missing-value handling was included as a no-imputation reference baseline, enabling direct comparison between imputation-based and imputation-free prediction strategies. Each experiment was repeated 5 times per imputation method and missingness level to account for variability due to random seed effects. Results were summarized using mean and standard deviation.

### Evaluation metrics

In this study, we employed several commonly used evaluation metrics to measure the performance of the imputation methods, with focus on root mean squared error (RMSE) and area under the receiver operating characteristic curve (AUC), which are key indicators of imputation performance. RMSE was used to evaluate the difference between imputed data and the original data, with smaller values indicating better imputation quality (i.e., the imputed data are closer to the true data) [24]. AUC was used to evaluate the performance of the imputed data in downstream classification tasks, especially its ability to predict binary classification tasks. Compared to RMSE, AUC places more emphasis on measuring whether the imputed data can provide effective discriminative power for the classification model [25]. Therefore, the comparison between RMSE and AUC helps provide a comprehensive understanding of the relationship between imputation quality and downstream task performance. For categorical variables present in the Breast Cancer and COVID-19 datasets, categorical accuracy was additionally reported as a type-aware evaluation metric, computed as the proportion of correctly recovered categories after decoding one-hot encoded imputed values back to their original categorical form, providing a more meaningful assessment of imputation quality for nonnumerical features compared to RMSE alone. Additionally, we used other supplementary metrics, such as mean absolute error, *R*-squared (*R*^2^), Kullback–Leibler divergence, Kolmogorov–Smirnov statistic (Kolmogorov–Smirnov test), and Wasserstein distance. These metrics offer a more detailed evaluation of imputation quality, particularly in terms of data distribution and feature differences. Furthermore, we ranked the RMSEs and AUCs of the 8 imputation methods under different missing rates. To assess whether observed differences in method performance were statistically significant, pairwise Wilcoxon signed-rank tests were conducted across all experimental conditions (datasets, mechanisms, and missingness rates). Bonferroni correction was applied to account for multiple comparisons, with a corrected significance threshold of *P* < 0.05. Results are reported in Tables S1 and S2 for RMSE and AUC comparisons, respectively. To assess the probability calibration of downstream predictions, Brier scores of the RF classifier are reported in Table S3. Across all datasets and missingness conditions, Brier scores ranged from 0.107 to 0.169, with most methods showing comparable calibration performance. GAIN exhibited slightly higher Brier scores in certain settings, particularly under MCAR, consistent with its higher variability in downstream classification performance. These results suggest that differences in probability calibration across imputation methods are generally modest in the evaluated clinical datasets.

## Results

### Traditional methods achieve lower RMSEs and more stable estimation quality

Figure 1 presents the RMSE values of 8 imputation methods across 3 datasets—MIMIC-III, Breast Cancer, and COVID-19—under MCAR, MAR, and MNAR mechanisms with missing rates ranging from 10% to 90%. Across all datasets and mechanisms, MICE and MissForest consistently show the lowest RMSE values, with limited variation as the missing rate increases, indicating robust and stable reconstruction performance across diverse missingness settings. Median imputation and SAITS generally occupy an intermediate position, with SAITS performing comparably to classical methods on the MIMIC-III dataset but showing higher variability on the COVID-19 dataset. In contrast, GAIN exhibits consistently higher RMSE values across all datasets and mechanisms, with substantial fluctuation and large standard deviations indicating imputation instability. This instability was particularly pronounced on the COVID-19 dataset under MAR, where GAIN’s RMSE continued to increase with higher missing rates rather than stabilizing. Similarly, MIWAE displays considerable volatility across mechanisms and datasets. GRU-D demonstrated relatively competitive RMSEs on the MIMIC-III dataset, which consists solely of continuous numerical variables, but exhibited substantially higher and more variable RMSEs on the COVID-19 dataset. The elevated reconstruction errors of SAITS and GRU-D on certain datasets are consistent with their original design for time-series data, which limits their ability to exploit structural patterns in cross-sectional tabular settings. Overall, these results suggest that classical methods—particularly MICE and MissForest—provide more reliable reconstruction performance across the evaluated clinical datasets and missingness conditions, while deep-learning-based methods exhibit greater sensitivity to dataset characteristics and missingness mechanisms. Median+Indicator exhibits RMSE values closely comparable to those of standard median imputation across all datasets and mechanisms, as both methods apply the same median-based fill strategy for missing values. The appended indicator columns do not contribute to imputation reconstruction quality but are intended to provide additional predictive signals for downstream classifiers.

**Fig. 1.**
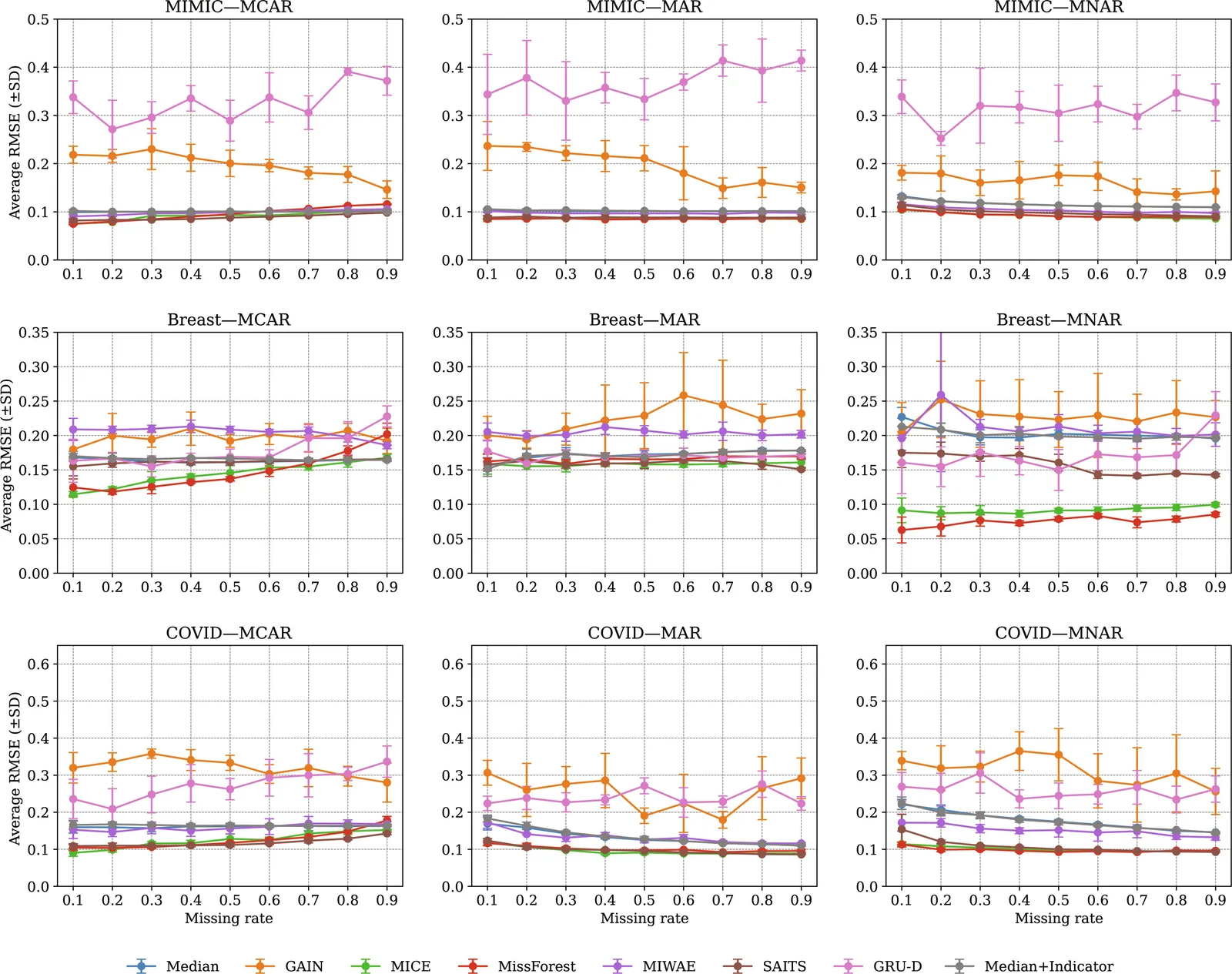
Root mean squared error (RMSE) values of 8 imputation methods across missing rates (10% to 90%) under missing completely at random (MCAR), missing at random (MAR), and missing not at random (MNAR) mechanisms in the MIMIC-III (Medical Information Mart for Intensive Care III), Breast Cancer, and COVID-19 datasets.

### Imputation methods show dataset- and classifier-dependent downstream performance

Figure 2 presents the downstream classification performance (RF AUC) of 8 imputation methods across 3 datasets under MCAR, MAR, and MNAR mechanisms, with XGBoost native missing-value handling included as a no-imputation reference baseline (dashed line).

**Fig. 2.**
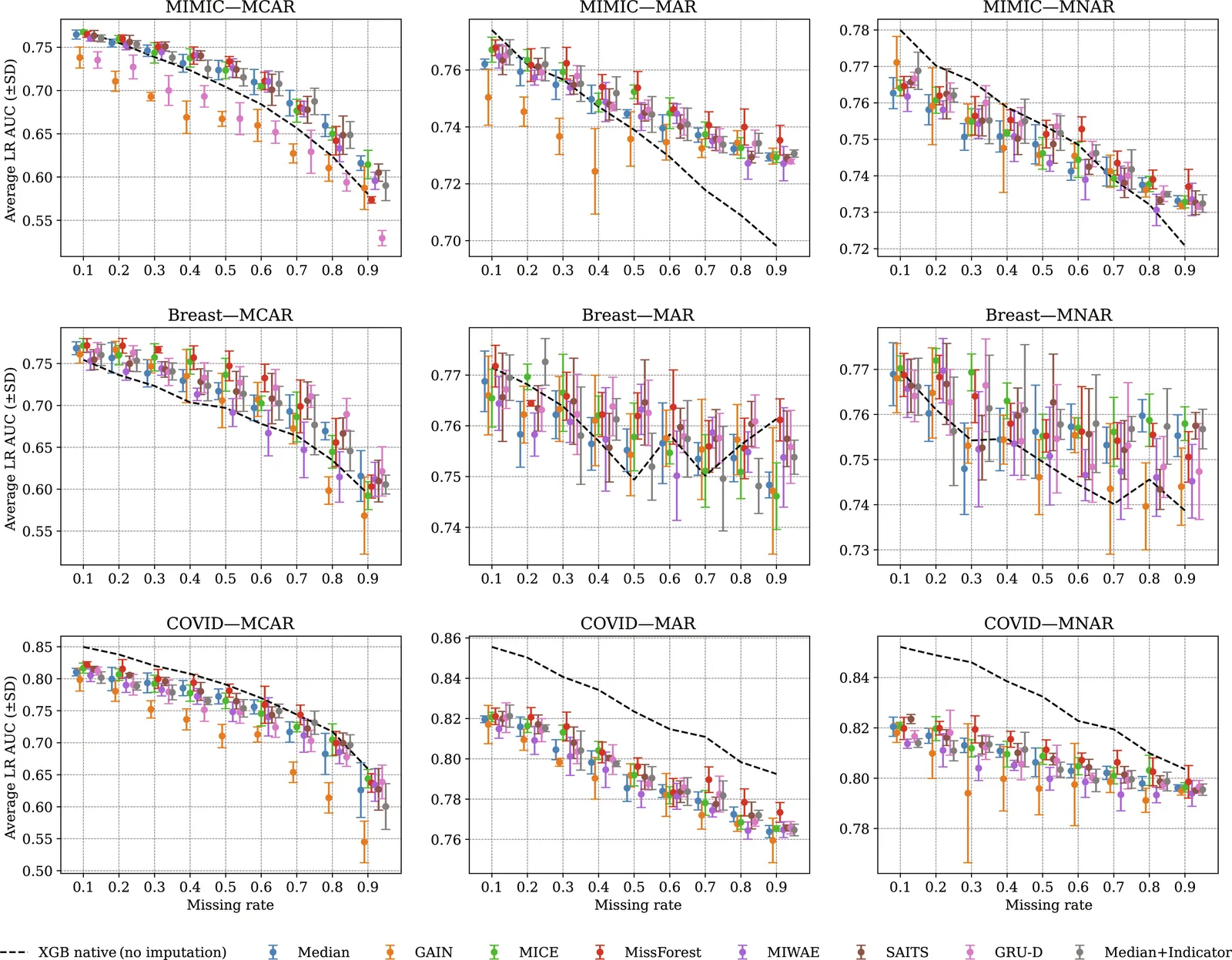
Random forest (RF) area under the receiver operating characteristic curve (AUC) values of 8 imputation methods across missing rates (10% to 90%) under the missing completely at random (MCAR), missing at random (MAR), and missing not at random (MNAR) mechanisms in the MIMIC-III (Medical Information Mart for Intensive Care III), Breast Cancer, and COVID-19 datasets. The dashed line represents XGBoost with native missing-value handling as a no-imputation reference baseline.

Across most settings, imputation-based methods outperform the XGBoost native baseline, particularly at lower missingness rates, suggesting that explicit imputation generally provides predictive benefit. However, this advantage diminishes at higher missingness rates, and in certain settings—notably the COVID-19 dataset under MAR and MNAR mechanisms—XGBoost native handling achieved competitive or higher AUC than most imputation-based methods, suggesting that model-native missing-value handling may be preferable in some clinical scenarios.

When comparing imputation methods, Median+Indicator generally achieves competitive or modestly higher AUCs compared to standard median imputation across most settings, particularly on the COVID-19 dataset, suggesting that the appended missingness indicator variables can provide useful predictive signals for downstream classifiers. However, the improvement over plain median imputation is not consistent across all datasets and mechanisms, indicating that the informativeness of missingness patterns varies with dataset characteristics. MICE and MissForest generally achieve competitive AUC values across datasets and mechanisms, while GAIN exhibits the most pronounced variability, particularly at higher missingness rates, frequently falling below the no-imputation baseline on the MIMIC-III dataset under MCAR. MIWAE shows lower and more variable AUCs on the Breast Cancer dataset, especially under the MAR and MNAR mechanisms. SAITS and GRU-D perform comparably to classical methods in some settings but show higher variability, consistent with their limited adaptability to cross-sectional tabular data.

To assess whether these patterns are classifier dependent, we additionally evaluated all methods using LR as a downstream classifier. Results are provided in Fig. S1. While absolute AUC values differed between RF and LR, the overall trend was largely consistent, with the exception that method rankings showed more notable variation under LR—for instance, GAIN consistently ranked last under LR across most settings, whereas its RF ranking was less stable. These findings suggest that downstream classifier choice can influence the relative ordering of imputation methods and should be considered alongside imputation strategy selection.

Across all mechanisms, AUC generally decreases as the missingness rate increases, with the decline being most pronounced under MCAR. Under MAR and MNAR, the decline is more gradual.

### Inconsistency between imputation accuracy and downstream predictive performance

Figure 3 presents the RMSE and RF AUC rankings of 8 imputation methods on the Breast Cancer dataset across 3 missingness mechanisms and missing rates from 10% to 90%. Rankings range from 1 (best) to 8 (worst).

**Fig. 3.**
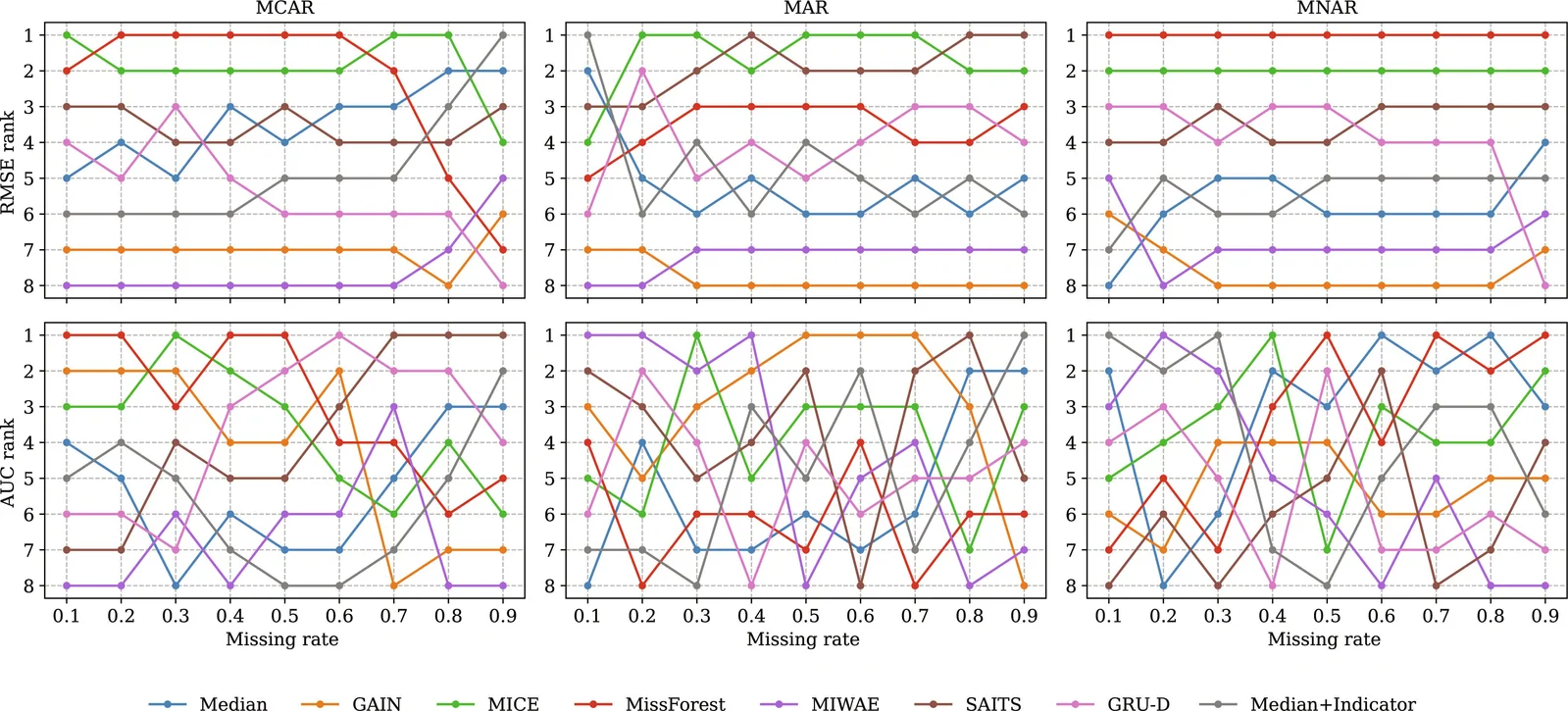
Root mean squared error (RMSE) ranking (top row) and random forest (RF) area under the receiver operating characteristic curve (AUC) ranking (bottom row) of 8 imputation methods on the Breast Cancer dataset under the missing completely at random (MCAR), missing at random (MAR), and missing not at random (MNAR) mechanisms across missing rates from 10% to 90%. Rankings range from 1 (best) to 8 (worst).

RMSE rankings show a relatively stable pattern across mechanisms and missing rates: MissForest and MICE consistently occupy the top 2 positions, while GAIN and MIWAE consistently rank at the bottom. Median+Indicator ranks closely alongside standard median imputation in RMSE across all mechanisms, consistent with their identical fill strategy. This stability suggests that reconstruction quality is relatively predictable given the method choice.

In contrast, AUC rankings exhibit substantially greater instability, with frequent rank crossings across all 3 mechanisms. No single method consistently dominates in downstream classification performance. Median+Indicator generally ranks comparably to or slightly above standard median imputation in AUC, with modest improvements observed particularly under the MAR and MNAR mechanisms, suggesting that missingness indicator variables can provide marginal but inconsistent predictive benefit. For instance, MissForest and MICE, despite their strong RMSE rankings, show considerable variation in AUC rankings across different missing rates and mechanisms. Conversely, GAIN, which consistently ranks last in RMSE, achieves relatively competitive AUC rankings in certain settings. These observations highlight a systematic decoupling between imputation reconstruction quality and downstream predictive utility, suggesting that RMSE alone is an insufficient criterion for imputation method selection in clinical prediction tasks.

Statistical comparisons using the Wilcoxon signed-rank test with Bonferroni correction (Tables S1 and S2) confirmed that MICE and MissForest did not differ significantly from each other in either RMSE or downstream AUC (*P* > 0.05 after correction), while both methods showed statistically significant advantages over GAIN in RMSE. In AUC comparisons, fewer statistically significant differences were observed across methods, with MICE, MissForest, SAITS, GRU-D, Median+Indicator, and median showing no significant pairwise differences, consistent with the high variability in downstream classification rankings noted above.

### Classical methods achieve higher categorical imputation accuracy

Figure 4 presents the categorical imputation accuracy for the Breast Cancer and COVID-19 datasets across 3 missingness mechanisms and missing rates from 10% to 90%.

**Fig. 4.**
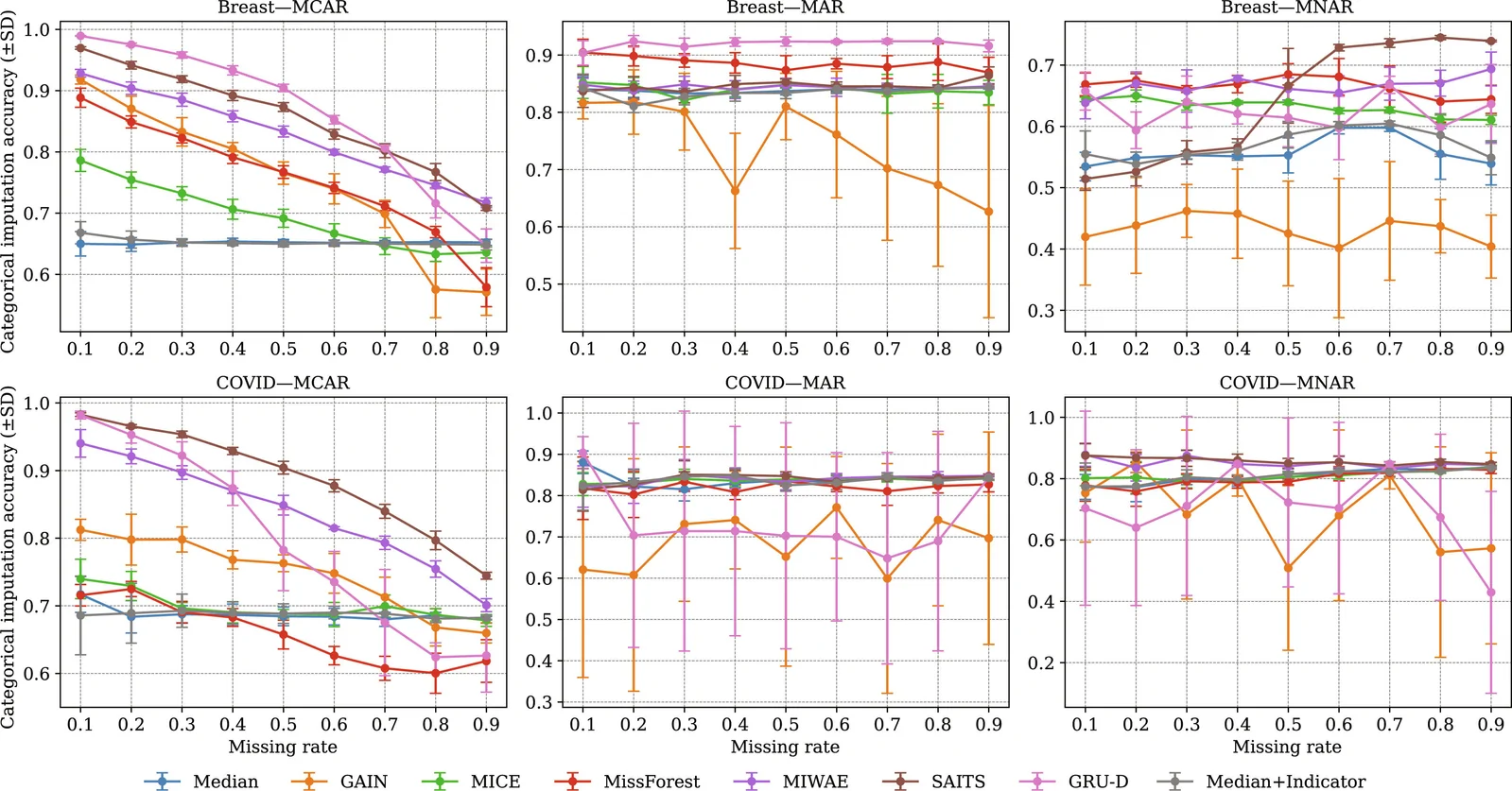
Categorical imputation accuracy of 8 imputation methods across missing rates (10% to 90%) under the missing completely at random (MCAR), missing at random (MAR), and missing not at random (MNAR) mechanisms in the Breast Cancer and COVID-19 datasets.

On the Breast Cancer dataset, MissForest and MICE consistently achieve the highest categorical accuracy across all mechanisms, with values generally remaining above 0.85 under the MAR and MNAR mechanisms. In contrast, GAIN exhibits the lowest and most variable categorical accuracy, with values dropping below 0.5 under MAR and MNAR at higher missing rates, and large standard deviations indicating substantial instability across repeated runs. Median imputation shows relatively lower categorical accuracy compared to other methods, reflecting its inability to capture interfeature dependencies when recovering categorical variables. Median+Indicator exhibits categorical imputation accuracy closely comparable to that of standard median imputation across all datasets and mechanisms, as the appended indicator columns do not alter the underlying median-based fill values and thus do not affect categorical reconstruction quality.

On the COVID-19 dataset, the pattern differs somewhat across mechanisms. Under MCAR, MIWAE and SAITS achieve notably high categorical accuracy, suggesting that these methods can effectively recover categorical structure when missingness is completely random. However, under the MAR and MNAR mechanisms, MICE and MissForest again demonstrate more stable performance, while GAIN and GRU-D show greater variability.

Overall, these results indicate that categorical imputation accuracy is more sensitive to the missingness mechanism than numeric RMSE is and that the relative advantage of classical methods over deep-learning-based approaches is particularly pronounced for categorical variable recovery under nonrandom missingness conditions.

### Joint visualization reveals that traditional methods consistently balance imputation accuracy and classification performance

To provide an overview of imputation performance under varying missing data conditions, we visualized the results of 8 imputation methods across 3 clinical datasets (Fig. 5). Each circle represents a method’s performance under a specific combination of missing mechanism and missing rate. The size of each bubble corresponds to the average RMSE (with smaller bubbles indicating lower imputation error), while the color intensity reflects the average AUC (with warmer colors representing higher classification performance).

**Fig. 5.**
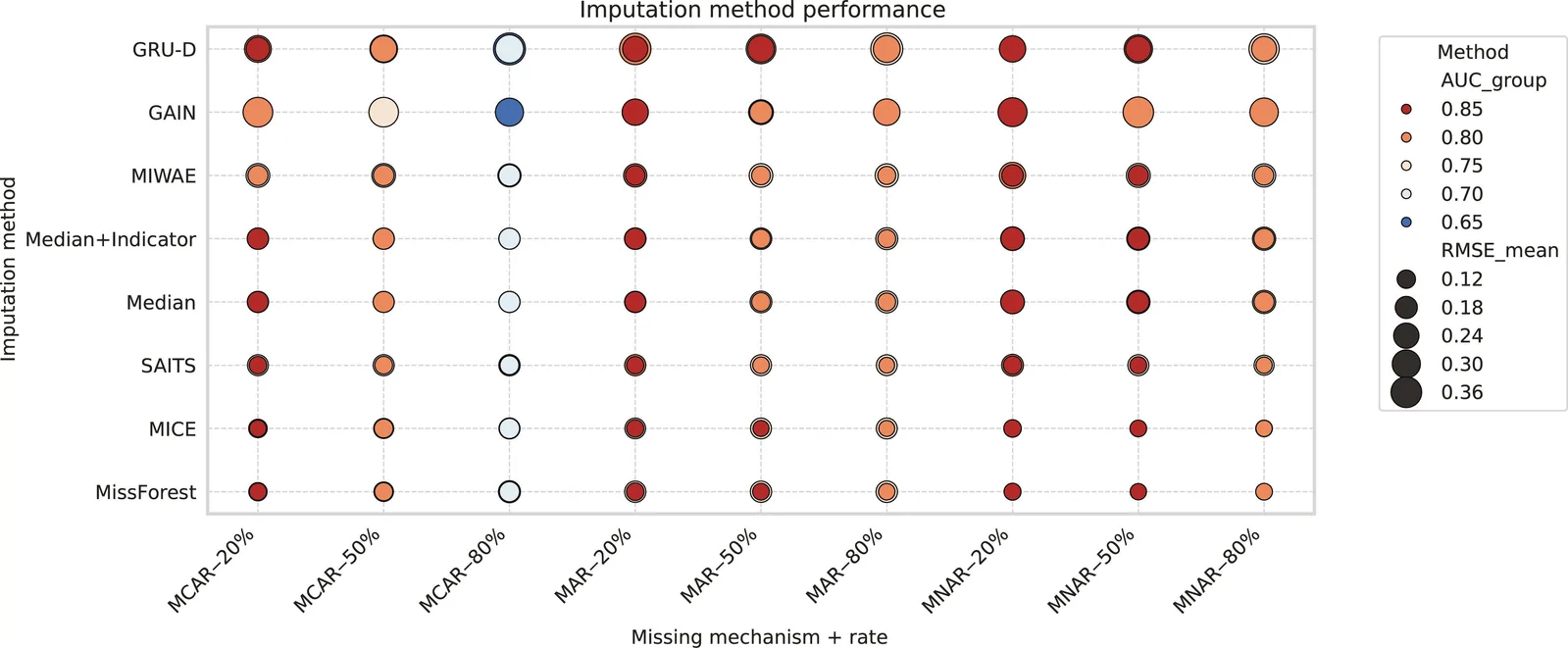
Comparison of 8 imputation methods across 3 clinical datasets under different missingness mechanisms and rates. Each circle represents a method’s performance under a specific condition. Bubble size indicates average root mean squared error (RMSE; larger size = higher error), and color intensity reflects average area under the receiver operating characteristic curve (AUC; darker red = better classification performance).

Across the datasets, traditional methods such as MICE and MissForest frequently demonstrated smaller bubble sizes and consistently warm colors, indicating lower imputation error and favorable downstream classification performance across most conditions. In contrast, deep-learning-based methods, particularly GAIN and MIWAE, showed more variable results depending on the missing data mechanism and rate. GAIN in particular exhibits larger bubble sizes across most settings, reflecting its consistently higher RMSE, despite achieving competitive AUCs in certain scenarios such as MCAR with low missingness. Median+Indicator displays bubble sizes and color intensities closely comparable to those of standard median imputation across all conditions, consistent with their shared fill strategy and similar downstream classification performance. SAITS demonstrates moderate bubble sizes in most settings, while GRU-D shows larger variability particularly under the MAR and MNAR mechanisms. These observations are consistent with the patterns identified in the preceding analyses and further highlight the difficulty of simultaneously achieving low reconstruction error and high downstream predictive performance across diverse clinical missingness conditions.

Regarding computational cost, MissForest required a substantially longer training time than other methods, particularly on the MIMIC-III dataset (approximately 700 s per run), attributable to its iterative RF fitting procedure on a larger sample size. In contrast, median imputation was the fastest method across all datasets, while GAIN and MIWAE required moderate training time. Median+Indicator exhibited training time comparable to that of standard median imputation, as the additional indicator columns introduce negligible computational overhead. Full runtime results are provided in Fig. S2. Based on profiling on the Breast Cancer dataset, peak memory consumption across methods ranged from approximately 3,100 to 3,600 MB, with no substantial differences observed between classical and deep-learning-based approaches, suggesting that memory overhead is not a primary distinguishing factor among the evaluated methods. A comprehensive memory analysis across all datasets remains a direction for future work.

## Discussion

In this study, we systematically evaluated the performance of 8 imputation methods—median, Median+Indicator, MICE, MissForest, GAIN, MIWAE, SAITS, and GRU-D—across 3 types of missing data mechanisms (MCAR, MAR, and MNAR) with a range of missing rates in 3 datasets (MIMIC-III, Breast Cancer, and COVID-19). Our goal was to understand how different imputation strategies affect both the accuracy of value imputation and the performance of downstream tasks and to examine how different imputation strategies relate to imputation accuracy and downstream performance under a range of missingness scenarios.

Traditional imputation methods, particularly MICE and MissForest, exhibited relatively stable performance across the evaluated datasets and missingness settings. Within our experimental setup, these methods generally achieved favorable rankings in terms of lower RMSEs and competitive AUC scores, including under more challenging conditions such as higher missingness rates and MNAR mechanisms. These observations are consistent with prior evaluations [17,26], which reported that regression- and tree-based imputers often provide robust performance on structured datasets. In contrast to some earlier benchmarks [27], our results suggest that MICE and MissForest can remain competitive under higher missingness levels and nonrandom mechanisms in the datasets considered. This robustness may be partly attributable to the inductive biases of these methods, such as linear or tree-based dependency modeling between features, which can be beneficial when information is incomplete [28–30]. In addition, their performance distributions across repeated experiments tended to exhibit lower variance, indicating more stable behavior across different missingness mechanisms and datasets. Among the newly included deep learning methods, SAITS demonstrated moderate reconstruction performance on the MIMIC-III dataset but showed higher variability on the COVID-19 dataset, while GRU-D exhibited competitive RMSE on purely numerical data but struggled on mixed-type datasets. These observations are consistent with the time-series-oriented design of both methods, which limits their adaptability to cross-sectional clinical tabular settings.

In contrast, deep-learning-based methods such as GAIN and MIWAE exhibited greater variability in their performance across the evaluated settings, particularly with respect to AUC under higher missingness rates. This variability may be related to their reliance on complex neural architectures, which can be more sensitive to limited sample sizes, sparsity, or irregular missingness patterns. While prior studies have shown that GAIN can outperform traditional methods under specific conditions [31,32], our results indicate that such performance advantages are not consistently observed across different datasets, missingness mechanisms, and missingness levels in our experiments. In addition, whereas earlier evaluations have demonstrated strong results for deep-learning-based imputers on synthetic or fully numerical datasets [8], our findings suggest that their empirical behavior can be more variable when applied to heterogeneous clinical tabular data. Rather than contradicting previous work, this observation highlights the importance of evaluating imputation methods within the data regimes and missingness configurations for which they are intended.

A key insight from our ranking analysis is the decoupling between imputation accuracy and downstream task performance. Across multiple settings, we observed that methods achieving lower RMSEs did not necessarily yield higher AUCs, while approaches with relatively larger reconstruction errors could still attain competitive or even superior classification performance. This observation challenges the common assumption that pointwise imputation accuracy alone is sufficient for downstream modeling and highlights the importance of evaluating imputation methods in terms of their task-level utility. Our findings are closely aligned with prior work by Shadbahr et al. [17], which critically examined the limitations of conventional imputation evaluation practices and demonstrated that improvements in imputation quality do not always translate into downstream performance gains. Their study provided important motivation for incorporating task-oriented evaluation criteria in benchmark studies. Building on this perspective, our contribution lies in providing a complementary empirical characterization of this discrepancy through a unified ranking-based comparison across both imputation and predictive metrics. Rather than focusing on individual datasets or methods, our analysis offers a systematic view of how discrepancies between RMSEs and AUCs manifest across datasets, missingness mechanisms, and imputation approaches, thereby reinforcing the need for multicriteria evaluation in imputation benchmarks. Furthermore, our analysis of downstream performance across 2 classifier families—RF and LR—revealed that method rankings were not always consistent across classifiers. For instance, GAIN ranked competitively under RF in certain settings but consistently ranked last under LR, suggesting that the choice of downstream classifier can itself influence the relative ordering of imputation methods. This finding further supports the use of multiple downstream models in imputation benchmarks, rather than relying on a single classifier.

Moreover, we found that the location of missingness played an important role in determining downstream predictive performance. Specifically, missing values occurring in more informative features led to a substantially larger degradation in classification performance than missingness in less informative variables, even when the overall imputation error (e.g., RMSE) remained moderate. This observation suggests that imputation quality cannot be fully characterized by aggregate reconstruction metrics alone, as errors in critical features may disproportionately affect downstream models. This heterogeneous sensitivity was more pronounced for deep-learning-based imputers such as GAIN and MIWAE, which exhibited larger performance fluctuations when key features were missing. While our study does not explicitly model feature importance during imputation, these results highlight the potential value of importance-aware or task-informed imputation strategies, particularly in clinical prediction settings where a small subset of variables often dominates model decisions.

We further observed that imputation performance varied substantially across datasets with different structural characteristics. On the MIMIC dataset, which predominantly consists of continuous variables, traditional methods such as MICE and MissForest exhibited consistently strong performance in both imputation accuracy and downstream classification. In contrast, on the Breast Cancer dataset—characterized by a higher proportion of categorical features and a relatively smaller sample size—MIWAE showed lower and more variable AUC performance, particularly under the MAR and MNAR mechanisms. While MIWAE has demonstrated effectiveness in prior studies involving numerical datasets with low-dimensional latent representations [9], our results suggest that its performance may be more sensitive to dataset composition and variable types commonly encountered in clinical tabular data.

Differences across missingness mechanisms further highlight the context-dependent nature of imputation behavior. Under MCAR, we observed a clearer separation in method rankings, with certain deep-learning-based approaches exhibiting higher variability. Although MNAR is often regarded as the most challenging setting due to its dependence on unobserved factors [33], our findings indicate that instability can also arise under MCAR, where missingness locations lack structured patterns. This suggests that performance variability may stem not only from the complexity of the missingness mechanism itself but also from the degree to which models can exploit—or fail to exploit—regularities in missingness structure. Among the augmented classical baselines, Median+Indicator demonstrated downstream classification performance generally comparable to or marginally higher than that of standard median imputation, particularly under the MAR and MNAR mechanisms where missingness patterns may carry predictive information. However, the improvement was modest and inconsistent across datasets, suggesting that the informativeness of missingness indicators varies with dataset characteristics. These results are consistent with prior observations that simple missingness indicators can provide incremental predictive benefit in certain clinical settings, while their utility remains context dependent.

Taken together, these results suggest that no single imputation strategy uniformly dominates across datasets, mechanisms, and missingness levels. While recent work has highlighted the flexibility of deep-learning-based imputers, traditional methods such as MICE and MissForest remained among the most consistently top-ranked approaches in our experiments, offering robust performance across diverse settings. At the same time, deep models like GAIN demonstrated competitive performance in specific scenarios—for example, on the COVID-19 dataset under MCAR at high missingness rates—indicating their potential effectiveness when data characteristics and dependency structures are favorable. These observations reinforce the importance of aligning imputation strategy selection with dataset properties and analytical objectives, rather than assuming universal method superiority.

Several limitations of this study should be acknowledged. First, all missingness in our experiments was artificially simulated from originally complete datasets, as ground-truth values are required for quantitative evaluation metrics such as RMSE. While this is standard practice in imputation benchmarking, simulated missingness—particularly MNAR—may not fully capture the complex, outcome-dependent patterns underlying real clinical data collection processes. The MNAR mechanism in our study was generated using pyampute with default parameterization, which represents a mathematical approximation and may not reflect clinically realistic MNAR scenarios such as selective laboratory test ordering or physician-driven measurement decisions. Second, the datasets used in this study are relatively modest in scale, and findings may not generalize to larger or more heterogeneous clinical populations. The MIMIC-III cohort was further restricted to a subset of patients meeting specific inclusion criteria, which may introduce selection bias. Third, our benchmark focuses exclusively on cross-sectional tabular data; the findings may not extend to longitudinal or time-series electronic health record settings, which present distinct missingness challenges. Fourth, all 3 datasets were processed and evaluated within a single analytical framework without external validation on independent hospital datasets, limiting the generalizability of our conclusions to other clinical environments. Fifth, while we include representative classical and deep learning imputation methods, several recent approaches—including diffusion-based imputers and transformer-based models—were not evaluated, and future benchmarks should examine these methods as they mature. Additionally, the time-series-based methods (SAITS and GRU-D) were adapted for cross-sectional use through pseudo-time-step construction, which may not fully reflect their intended use cases and could underestimate their potential in appropriate temporal settings.

Finally, downstream evaluation was based on discriminative performance (AUC); calibration and fairness across patient subgroups were not formally assessed and represent important directions for future work. Additionally, categorical imputation evaluation was limited to point-estimate accuracy metrics; probabilistic evaluation metrics such as log-loss were not included, as several methods in the benchmark do not natively output categorical probability distributions, precluding a consistent cross-method comparison. Future work may address this through methods that natively support probabilistic categorical imputation.

These findings carry practical implications for clinical decision support. In high-stakes settings such as ICU mortality prediction, the choice of imputation strategy can meaningfully affect model outputs, particularly when missingness occurs in clinically informative variables such as vital signs or laboratory values. Our results suggest that relying solely on reconstruction metrics such as RMSE to select an imputation method may be insufficient; task-oriented evaluation is essential before deploying imputed data in clinical pipelines. Furthermore, imputation quality may vary systematically across patient subgroups—for instance, patients with more severe conditions may have higher rates of missing measurements, potentially introducing bias into downstream predictions. While a formal fairness analysis is beyond the scope of this benchmark, future work should examine whether imputation errors disproportionately affect vulnerable subgroups. Additionally, beyond predictive accuracy (AUC), calibration of downstream models trained on imputed data warrants attention, as poorly calibrated probability estimates can mislead clinical decision-making even when discrimination metrics appear acceptable.

## Conclusion

In this study, we benchmarked 8 imputation methods—median, Median+Indicator, MICE, MissForest, GAIN, MIWAE, SAITS, and GRU-D—across 3 real-world clinical tabular datasets under 3 missingness mechanisms (MCAR, MAR, and MNAR) and missing rates ranging from 10% to 90%, evaluating both imputation accuracy and downstream predictive utility across multiple classifiers.

Within the scope of this benchmark, classical methods—particularly MICE and MissForest—demonstrated consistently robust performance across the evaluated settings, while deep-learning-based methods exhibited greater sensitivity to dataset characteristics and missingness conditions. For datasets containing categorical variables, classical methods also maintained higher categorical imputation accuracy, particularly under nonrandom missingness mechanisms. Notably, XGBoost with native missing-value handling achieved competitive or superior downstream performance in certain settings, indicating that explicit imputation does not always provide predictive benefit over model-native strategies.

A key finding was the systematic decoupling between RMSE-based reconstruction quality and downstream predictive utility, consistently observed across multiple classifiers and datasets. Method rankings under RF and LR showed notable variation in certain settings, further suggesting that downstream classifier choice should be considered alongside imputation strategy selection. These results highlight the importance of task-oriented, multicriteria evaluation when selecting imputation strategies for clinical tabular data and underscore that no single method universally dominates across all settings examined in this study.

## Ethical Approval

This study used only de-identified, publicly available datasets and did not involve any direct interaction with human participants. The MIMIC-III and Breast Cancer datasets were obtained from previously published studies that had received Institutional Review Board (IRB) approval and released preprocessed, anonymized subsets for secondary research use [17]. The COVID-19 dataset was downloaded from the Kaggle COVID-19 Data Project (https://www.kaggle.com/datasets/S%C3%ADrio-Libanes/covid19), which contains fully anonymized clinical data. As such, our study was exempt from additional IRB approval, and no individual consent was required.

## Data Availability

The MIMIC-III dataset is available via PhysioNet (https://physionet.org/content/mimiciii/1.4/) after completing the required training and data use agreement. In this study, we used a preprocessed version of the MIMIC-III dataset provided by Shadbahr et al. [17] as part of their published work. The Breast Cancer dataset used in this study is a processed subset originally derived from data at MSKCC, made available by Shadbahr et al. [17]. The original raw data are not publicly available due to patient privacy, but we used the processed version provided by the authors for research use. The COVID-19 dataset used in this study is publicly available from Kaggle at https://www.kaggle.com/datasets/S%C3%ADrio-Libanes/covid19/data. Access requires a Kaggle account and agreement to the dataset’s terms of use. The code will be made publicly available upon publication at https://github.com/jokerxuez/a1.
