# Supplementary material for: Comparison of Imputation Strategies for Incomplete Electronic Health Data
Source: Health Data Sci. 2026 Nov 2;6:0493. doi: 10.34133/hds.0493 (PMC13724834; doi:10.34133/hds.0493)
Supplement: Supplementary file 1 [file hds.0493.f1.pdf]

Supplementary Materials

Comparison of Imputation Strategies for Incomplete Electronic Health Data

Supplementary Figure

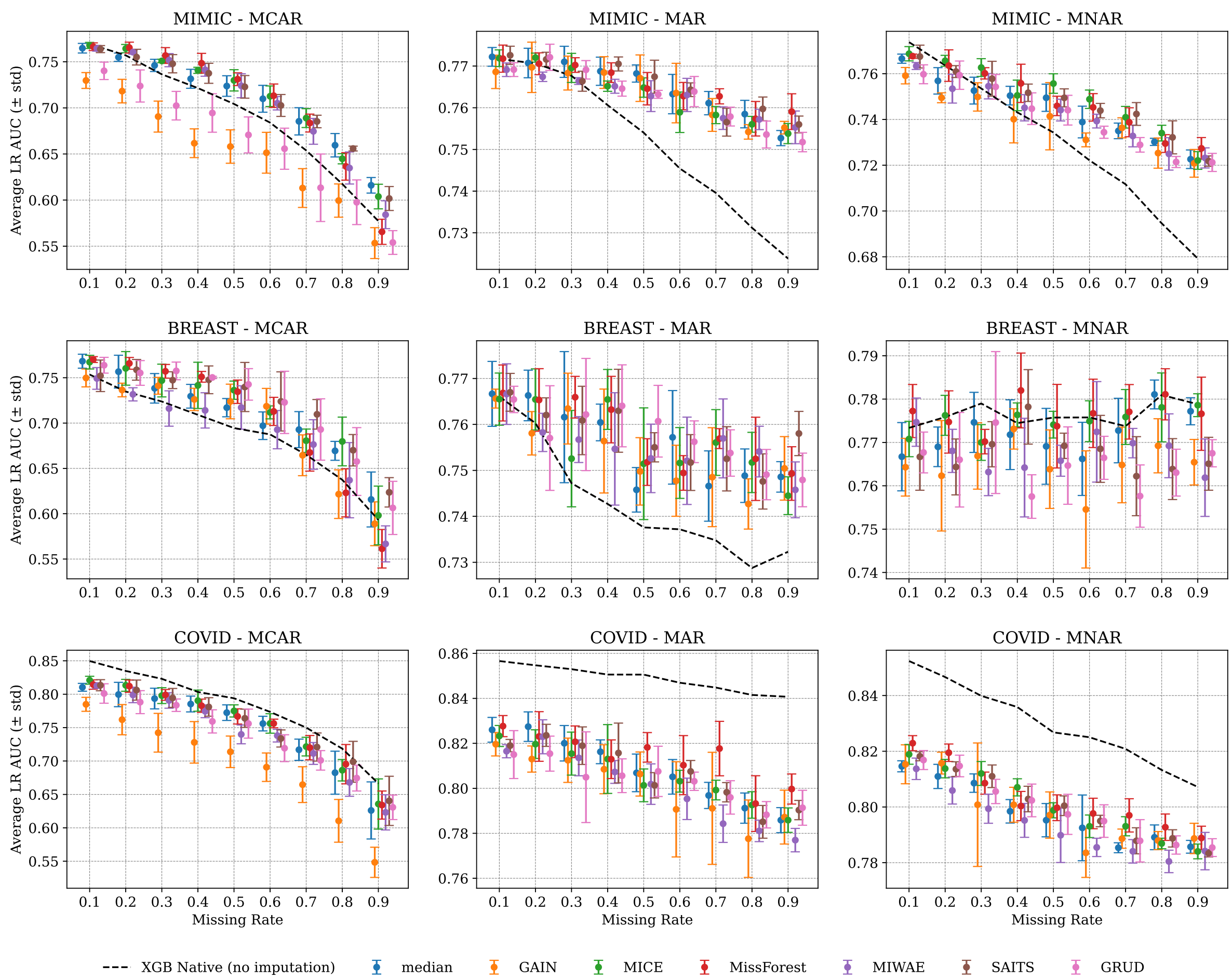

Figure S1: LR AUC values of seven imputation methods across missing rates (10% to 90%) under MCAR, MAR, and MNAR mechanisms in the MIMIC-III, Breast Cancer, and COVID-19 datasets. The dashed line represents XGBoost with native missing value handling as a no-imputation reference baseline.

Supplementary Table S3 presents the Brier scores of the Random Forest classifier trained on imputed data across all datasets, missingness mechanisms, and methods. Brier score measures the mean squared difference between predicted probabilities and true binary outcomes, with lower values indicating better probability calibration.

Table 1: Pairwise Wilcoxon signed-rank test results (Bonferroni-corrected p-values) for RMSE comparisons across all eight imputation methods. * indicates statistical significance ($p < 0.05$ after correction); ns indicates non-significant.

	Median	GAIN	MICE	MissForest	MIWAE	SAITS	GRUD	Median+Ind.
**Median**	–	<0.001*	<0.001*	<0.001*	1.000ns	<0.001*	<0.001*	0.885ns
**GAIN**	<0.001*	–	<0.001*	<0.001*	<0.001*	<0.001*	1.000ns	<0.001*
**MICE**	<0.001*	<0.001*	–	1.000ns	<0.001*	0.002*	<0.001*	<0.001*
**MissForest**	<0.001*	<0.001*	1.000ns	–	<0.001*	1.000ns	<0.001*	<0.001*
**MIWAE**	1.000ns	<0.001*	<0.001*	<0.001*	–	<0.001*	<0.001*	1.000ns
**SAITS**	<0.001*	<0.001*	0.002*	1.000ns	<0.001*	–	<0.001*	<0.001*
**GRUD**	<0.001*	1.000ns	<0.001*	<0.001*	<0.001*	<0.001*	–	<0.001*
**Median+Ind.**	0.885ns	<0.001*	<0.001*	<0.001*	1.000ns	<0.001*	<0.001*	–

Table 2: Pairwise Wilcoxon signed-rank test results (Bonferroni-corrected p-values) for AUC comparisons across all eight imputation methods. * indicates statistical significance ($p < 0.05$ after correction); ns indicates non-significant.

	Median	GAIN	MICE	MissForest	MIWAE	SAITS	GRUD	Median+Ind.
**Median**	–	<0.001*	1.000ns	1.000ns	0.443ns	1.000ns	1.000ns	1.000ns
**GAIN**	<0.001*	–	<0.001*	<0.001*	0.052ns	<0.001*	<0.001*	<0.001*
**MICE**	1.000ns	<0.001*	–	1.000ns	0.015*	1.000ns	0.128ns	1.000ns
**MissForest**	1.000ns	<0.001*	1.000ns	–	0.025*	1.000ns	0.023*	1.000ns
**MIWAE**	0.443ns	0.052ns	0.015*	0.025*	–	1.000ns	1.000ns	0.041*
**SAITS**	1.000ns	<0.001*	1.000ns	1.000ns	1.000ns	–	1.000ns	1.000ns
**GRUD**	1.000ns	<0.001*	0.128ns	0.023*	1.000ns	1.000ns	–	0.024*
**Median+Ind.**	1.000ns	<0.001*	1.000ns	1.000ns	0.041*	1.000ns	0.024*	–

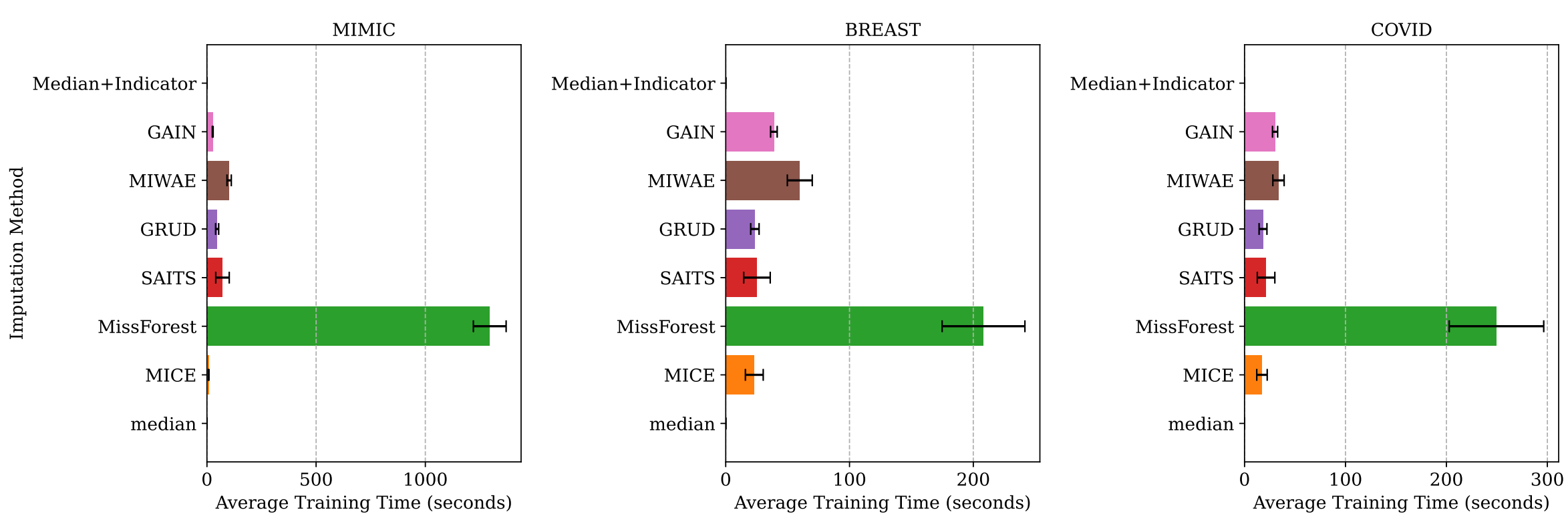

Figure S2: Average training time (seconds) of seven imputation methods across three clinical datasets (MIMIC-III, Breast Cancer, and COVID-19). Error bars indicate standard deviation across repeated runs. MissForest required substantially longer training time on the MIMIC-III dataset due to its larger sample size and iterative random forest fitting procedure.

Table 3: Brier scores (mean ± std) of the Random Forest classifier across datasets, missingness mechanisms, and imputation methods. Lower values indicate better probability calibration.

**Dataset**	**Mechanism**	**Median**	**Median+Ind.**	**MICE**	**MissForest**	**MIWAE**	**SAITS**	**GRUD**	**GAIN**
3*MIMIC	MCAR	0.123±0.002	0.123±0.002	0.122±0.002	0.121±0.002	0.119±0.002	0.121±0.002	0.126±0.001	0.147±0.019
	MAR	0.112±0.001	0.111±0.001	0.112±0.001	0.111±0.001	0.113±0.001	0.112±0.001	0.112±0.001	0.133±0.015
	MNAR	0.107±0.001	0.107±0.001	0.107±0.001	0.107±0.001	0.108±0.001	0.108±0.001	0.108±0.001	0.119±0.012
3*BREAST	MCAR	0.164±0.005	0.161±0.004	0.157±0.003	0.158±0.004	0.159±0.003	0.155±0.003	0.154±0.003	0.156±0.003
	MAR	0.152±0.003	0.152±0.004	0.151±0.004	0.151±0.004	0.146±0.002	0.149±0.004	0.150±0.004	0.146±0.003
	MNAR	0.146±0.003	0.147±0.004	0.148±0.003	0.146±0.002	0.147±0.003	0.152±0.004	0.151±0.003	0.147±0.004
3*COVID	MCAR	0.148±0.005	0.149±0.005	0.147±0.004	0.148±0.005	0.152±0.005	0.148±0.004	0.150±0.005	0.169±0.010
	MAR	0.131±0.003	0.132±0.003	0.131±0.003	0.128±0.002	0.131±0.003	0.131±0.003	0.131±0.003	0.144±0.009
	MNAR	0.130±0.002	0.129±0.003	0.129±0.003	0.128±0.003	0.127±0.003	0.127±0.003	0.128±0.003	0.136±0.007
